# Nesting success of wood‐cavity‐nesting bees declines with increasing time since wildfire

**DOI:** 10.1002/ece3.5657

**Published:** 2019-10-02

**Authors:** Michael P. Simanonok, Laura A. Burkle

**Affiliations:** ^1^ Department of Ecology Montana State University Bozeman MT USA

**Keywords:** native bees, pollination services, pollinators, pyrodiversity

## Abstract

Bees require distinct foraging and nesting resources to occur in close proximity. However, spatial and temporal patterns in the availability and quantity of these resources can be affected by disturbances like wildfire. The potential for spatial or temporal separation of foraging and nesting resources is of particular concern for solitary wood‐cavity‐nesting bees as they are central‐place, short‐distance foragers once they have established their nest. Often the importance of nesting resources for bees have been tested by sampling foraging bees as a proxy, and nesting bees have rarely been studied in a community context, particularly postdisturbance. We tested how wood‐cavity‐nesting bee species richness, nesting success, and nesting and floral resources varied across gradients of wildfire severity and time‐since‐burn. We sampled nesting bees via nesting boxes within four wildfires in southwest Montana, USA, using a space‐for‐time substitution chronosequence approach spanning 3–25 years postburn and including an unburned control. We found that bee nesting success and species richness declined with increasing time postburn, with a complete lack of successful bee nesting in unburned areas. Nesting and floral resources were highly variable across both burn severity and time‐since‐burn, yet generally did not have strong effects on nesting success. Our results together suggest that burned areas may provide important habitat for wood‐cavity‐nesting bees in this system. Given ongoing fire regime shifts as well as other threats facing wild bee communities, this work helps provide essential information necessary for the management and conservation of wood‐cavity‐nesting bees.

## Graphical abstract

Bees require foraging and nesting resources which do not always co‐occur in space and time, and this can be exacerbated by disturbances like wildfire. We studied how wood‐cavity‐nesting bees respond to wildfire by looking at their nesting success across burn severity and time‐since‐burn. We observed nesting success to decline with greater time‐since‐burn, highlighting the importance of burned areas for wood‐cavity‐nesting bee habitat.

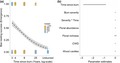

## 1 INTRODUCTION

Bees require access to preferred nesting and floral resources for survival and successful reproduction, yet these two resources do not always overlap in space and time (Westrich, 1996). This is particularly true for wood‐cavity‐nesting bees, as many of the characteristics associated with wood‐cavity‐nesting habitat, such as large standing tree snags, abundant coarse woody debris, or high canopy cover (Morato & Martins, 2006; Westerfelt, Widenfalk, Lindelow, Gustafsson, & Weslien, 2015), represent physical characteristics which may restrict floral abundance or diversity (Peterson, Reich, & Wrage, 2007; Potts et al., 2005). Additionally, most solitary bees are central‐place foragers, typically staying within a few hundred meters of their nests (Gathmann & Tscharntke, 2002) and are thus heavily influenced by local‐scale habitat factors (Hopfenmüller, Steffan‐Dewenter, & Holzschuh, 2014; Murray et al., 2012). This combination of short foraging distances from a central location and necessity for adequate nesting and floral resources makes the relative proximity, arrangement, quantity, and quality of bees' nesting and foraging habitats essential for nesting success (Westrich, 1996).

Disturbances, such as wildfire, can affect the quality, quantity, timing, and spatial distribution of bee nesting and floral resources across the landscape (Peralta, Stevani, Chacoff, Dorado, & Vazquez, 2017; Potts et al., 2005). Wildfire is a globally occurring ecological process and a natural part of many ecosystems, yet fire suppression and climate change continue to amplify the extent and severity of fires (Bowman et al., 2009). The homogenization of landscapes by high‐severity burns (Bowman et al., 2009) could strongly affect the presence and relative abundance of both nesting and foraging habitats for wood‐cavity‐nesting bees, creating landscapes where, for example, suitable nesting resources are either unavailable or too distant from adequate floral resources (Potts et al., 2010; Westrich, 1996). High‐severity burns generally have lower local species richness and site‐to‐site variation across the landscape in both bee and floral communities when compared to areas of greater pyrodiversity (Lazarina et al., 2019; Ponisio et al., 2016). By contrast, mixed‐severity burns with high pyrodiversity create a landscape pattern of high‐ to low‐severity patches, providing a variety of biological legacies (e.g., snags or coarse woody debris) and floral communities across the landscape (Arno & Fiedler, 2005). Additionally, the vegetation and biological legacies, including old solitary bee nesting cavities, that survive low‐severity burn patches are noted as important bee nesting resources (Brown, York, Christie, & McCarthy, 2017; Robinson et al., 2013). Wood‐cavity‐nesting bees depend on cavities created by wood‐boring beetles (Sydenham, Hausler, Moe, & Eldegard, 2016; Westerfelt et al., 2015), and beetle abundance and richness; thus, the number and sizes of the wood cavities available to bees can vary with burn severity as well as time‐since‐burn, with taxa‐specific positive or negative responses (Ray et al., 2019). Taken together, wood‐cavity‐nesting bees may be particularly vulnerable to the effects of high‐severity wildfires due to limited nesting or floral resources.

Generally, areas burned by wildfire benefit bees compared to unburned areas (Burkle, Simanonok, Durney, Myers, & Belote, 2019; Carbone, Tavella, Pausas, & Aguilar, 2019). However, most research on bee communities compares pre‐ to postburn conditions or focuses on the years immediately postburn, leaving an incomplete understanding of the effects of wildfire, as well as burn severity, on bees across successional time postburn. For the floral community, the effects of burn severity may develop, attenuate, or persist with time‐since‐burn (Abella & Fornwalt, 2015). Likewise, nesting resources associated with wood‐cavity‐nesting bees vary with time‐since‐burn, and changes in bee community composition has been associated with variable abundance and diversity of nesting resources postburn (Grundel et al., 2010; Potts et al., 2005), suggesting that nesting resource availability could affect both bee abundance and species richness with increasing time‐since‐burn. For example, in a Mediterranean pine forest system, the number of available wood cavities was highest in the years immediately postburn, while the amount of coarse woody debris peaked in older burns (ca. 16 years postburn, Potts et al., 2005). Thus, the availability of different nesting resources is not necessarily concurrent through succession. How these nesting resources correlate to bee abundance or richness is inconsistent as well. For example, in two different studies from the same ecosystem, wood‐cavity‐nesting bees were most abundant in older burns (20–28 years postburn, Lazarina et al., 2016), while bee abundance generally peaked in the years immediately postburn (Potts et al., 2003). There is also evidence that in a more xeric biome, wood‐cavity‐nesting bees recover quickly postburn, and diet‐generalist, wood‐cavity‐nesting species can dominate community composition in recently burned sites (Peralta et al., 2017).

Actively nesting bees are notoriously difficult to locate in their natural nesting habitat, particularly wood‐cavity‐nesting bees (Roulston & Goodell, 2011), and studies investigating their use of nesting resources have often used passive trapping via bee‐bowls (e.g., Grundel et al., 2010) or hand‐netting of foraging bees (e.g., Potts et al., 2005) to infer use of local bee nesting resources. One study used artificial nests postburn to investigate changes in foraging patterns and network structure (Peralta et al., 2017) but did not relate these patterns to any direct measurements of nesting resources. Furthermore, previous studies have primarily investigated broad community‐level metrics such as abundance and richness, without consideration of demographic properties which underlay those responses, such as nesting success or bee emergence, which can be important for understanding how bee populations respond to varied landscapes (e.g., Persson, Mazier, & Smith, 2018) such as those postdisturbance. Generally, the effects of nesting habitats and resources on wood‐cavity‐nesting bee populations and communities have not been adequately demonstrated independently of floral resources for wood‐cavity‐nesting bees (Roulston & Goodell, 2011).

We investigated how wood‐cavity‐nesting bee species richness and nesting success varied after wildfire, and how nesting and floral resource differences across a gradient of burn severity as well as how time‐since‐burn may affect the community of nesting bees and their nesting success. We placed bee nesting boxes in areas of mixed‐ and high‐severity burn within four wildfires spanning a time‐since‐burn gradient of 3–25 years post‐burn, including an “unburned” control, using a chronosequence approach (e.g., Hutto & Belote, 2013) to test: (a) how nesting and floral resources (i.e., coarse woody debris, wood cavity density, canopy cover, floral richness, and floral abundance) differ with burn severity and time‐since‐burn and (b) how species richness of nesting bees and the nesting success of wood‐cavity‐nesting bees vary with burn severity and time‐since‐burn, as well as how nesting and floral resources may affect those relationships.

## 2 MATERIALS AND METHODS

### 2.1 Study site

Four wildfires from the Absaroka Mountains of southwest Montana, USA, were selected to include a range of burn severities and time‐since‐burn (Table 1). Additionally, we selected an unburned area (i.e., no recorded burns in at least 75 years) located approximately 4 km from all other sites in this study and with similar topographical characteristics to the burned areas (Table 1). Our study areas within these burn perimeters were located on public lands administered by the US Forest Service within the Custer Gallatin National Forest and Absaroka‐Beartooth Wilderness. Wildfire is a natural ecosystem process in this region, and the study area consists of forests dominated by lodgepole pine (*Pinus contorta*) and Douglas fir (*Pseudotsuga menziesii*; Burkle, Myers, & Belote, 2015). The current fire regime is characterized by mixed‐severity burns with high‐severity events increasing in occurrence and fire return intervals of 10–80 years (US Department of Agriculture, Forest Service, & Missoula Fire Sciences Laboratory 2012).

**Table 1:** Name, location, ignition date, and average characteristics of sampling plots within burn perimeters

	Elevation (m)	Slope (°)	Aspect (°)	Severity (RdNBR)	Ignition	Area (acres)	Location
Emigrant							
High	1,901.7 ± 82.5	20.9 ± 6.2	102.8 ± 20.8	488.5 ± 115.2	Aug. 16, 2013	11,834	45.23°, −110.73°
Mixed	1,894.6 ± 45.6	16.3 ± 4.9	196.3 ± 6.4	138.2 ± 85.3			
Pine Creek							
High	1,961.7 ± 31.7	20.2 ± 3.7	79.3 ± 19.7	1,141.8 ± 23.4	Aug. 29, 2012	8,572	45.52°, −110.50°
Mixed	1,895.8 ± 47.4	23.0 ± 3.3	172.6 ± 16.9	770.3 ± 141.2			
Wicked Creek							
High	2,130.2 ± 12.5	24.2 ± 7.0	72.5 ± 34.2	1,068.0 ± 49.5	Aug. 8, 2007	28,674	45.26°, −110.47°
Mixed	2,087.1 ± 15.6	16.5 ± 4.4	116.0 ± 25.3	679.3 ± 100.9			
Thompson Creek							
High	2,151.3 ± 8.2	24.6 ± 2.2	155.2 ± 23.6	898.7 ± 49.8	Jul. 16, 1991	6,979	45.24°, −110.55°
Mixed	2,070.4 ± 4.1	33.8 ± 4.1	189.6 ± 27.0	448.8 ± 86.4			
Unburned	2,228.8 ± 43.9	12.8 ± 2.7	118.7 ± 6.0	–	–	–	45.25°, −110.41°

Note

Given latitude and longitude is an approximate centroid of study plot locations within each burn perimeter. Elevation, aspect, and slope are mean values with 95% confidence intervals across plots. Note that the Emigrant fire was managed as part of the Miners Paradise Complex and the Wicked Creek fire was managed with the WH Complex.

To compare bee nesting across burn severity, we selected two 15ha sampling blocks of high‐severity burn and two of mixed‐severity burn within each wildfire perimeter. Burn severity categories were determined by the Monitoring Trends in Burn Severity (MTBS) project (Eidenshink et al., 2007, Table 1) for all wildfires except Emigrant. Emigrant burn severity for site selection was determined from Burned Area Emergency Response data (BAER, Parsons, Robichaud, Lewis, Napper, & Clark, 2010) because MTBS data were not yet published for the Emigrant fire at the start of data collection in June 2014. Each block contained three plots. A mixed‐severity block was assigned one low‐severity, one moderate‐severity, and one high‐severity plot, while a high‐severity block was assigned three high‐severity plots. Locations for plots were selected using Generalized Random Tessellation Stratified Spatial Sampling, which accounts for the spatial distribution of plots to minimize clustering from true random selection and allowed us to stratify plots based on burn severity categories (Kincaid & Olsen, 2011). Mean nearest‐neighbor distance between plots within blocks was 240.6 m ± 20.7 *SE*. Relative difference Normalized Burn Ratio (RdNBR) values were extracted from MTBS fire perimeters at the plot‐level (Eidenshink et al., 2007) for all wildfires for use in analyses. We used RdNBR because it allows for informative analysis of local burn severity effects on wild bees (e.g., Galbraith, Cane, Moldenke, & Rivers, 2019) compared to categorical delineations (i.e., mixed‐ vs. high‐severity), and it also provides standardized quantification of burn severity across our chronosequence of burns.

### 2.2 Field sampling

Within each plot, a bee nesting box was affixed to the snag nearest to the center of the plot in early June 2016. When no standing snags were present, the tallest coarse woody debris (for 9 of 54 plots) or stump (for 5 of 54 plots) nearest to the center of the plot was used. Nest boxes were always placed with their cavity openings facing southeast, and approximately 1m from the ground (mean = 1.097, 95% CI ± 0.009). Nest boxes were constructed out of pine or poplar, and each box had 16 drilled cavities for cardboard bee nesting tubes. Four sizes of tubes were used in each box (3, 4, 5, and 6 mm diameter) to maximize the number of species which could potentially nest in them. Nest boxes were checked at least every other week from June through August 2016; occupied nesting tubes were removed and replaced with unused, empty tubes. Occupied tubes were then individually stored in plastic bottles with five 1.5 mm air holes and overwintered in the ambient conditions of an uninsulated shed in Bozeman, MT from September 2016 until emergence was first detected in April 2017. Once bees began to emerge, tubes were moved into room temperature lab conditions and checked twice per week for new emergence from April to August 2017. After emergence, bees were frozen and then identified to species using a combination of keys (Michener, McGinley, & Danforth, 1994; Sheffield, Ratti, Packer, & Griswold, 2011), regional experts (e.g., Reese, Burkle, Delphia, & Griswold, 2018) and a reference collection maintained by the Burkle Lab at Montana State University, where specimens from this study are kept. All bees for this study were identified to species save one individual, a male *Osmia* spp. which was considered a morphospecies. Full species list of bees which emerged from nesting tubes for each wildfire severity, in each wildfire perimeter, and collection date are available in Simanonok (2018). Because we did not overwinter bees for multiple years, our design may have underestimated the emergence of bees which require multiple overwintering periods (e.g., Forrest, Cross, & CaraDonna, 2019). We considered successful adult bee emergence as one or more adult bees emerging from an individual nesting tube, and we recorded nesting success as a binomial response of bee emergence at the individual nesting tube level. We also recorded the species richness of emerged nesting bee species per plot.

When nest boxes were placed at plots, habitat characteristics of each plot were sampled within a 2 m × 25 m band transect that was centered on each plot, perpendicular to the slope. Within the band transect, we recorded all coarse woody debris (CWD) as volume in m^3^/ha following Harmon and Sexton (1996). The number of wood cavities, defined as 3–6 mm diameter holes to match our nesting tube sizes, was recorded for all CWD, snags, and trees within the 2 × 25 m transect. Canopy photographs were taken from the center of the plot using a fish‐eye lens and canopy cover was calculated using Gap Light Analyzer (Frazer, Canham, & Lertzman, 1999). To census the floral community and record floral abundance and richness, all open flowers of each species were identified and counted every other week within the band transect at all plots except those in the mixed‐severity plots of the older burn, Thompson Creek (*n* = 6 plots, Table 1) due to logistical limitations.

### 2.3 Statistical analysis

We first assessed how nesting (i.e., wood cavities, coarse woody debris, and canopy cover) and floral resources (i.e., floral abundance and richness) differed across burn severity (RdNBR), time‐since‐burn (years postburn), and their interaction across plots using generalized linear models (GLM). Number of wood cavities, floral richness, and floral abundance are count responses which we analyzed with quasipoisson distributions due to overdispersion. Given previously published nonlinear trends in some of our parameters of interest across time‐since‐burn (e.g., Potts et al., 2003, 2005), we tested for such relationships in number of wood cavities, floral abundance, floral species richness, and bee species richness across time‐since‐burn by adding a nonlinear parameter for time‐since‐burn and performing a drop‐in‐deviance test comparing GLMs with and without the nonlinear parameter. Nonlinear parameters did not improve fit for number of wood cavities (*F*_48,50_ = 0.53, *p* = .60) or bee species richness (*F*_38,40_ = 1.24, *p* = .29), yet improved model fit for floral abundance (*F*_42,44_ = 4.80, *p* = .01) and floral richness (*F*_42,44_ = 6.97, *p* < .01); thus, those parameters were retained only for models testing floral abundance and richness.

To test for effects of burn severity (RdNBR), time‐since‐burn (years postburn), the interaction between burn severity and time‐since‐burn, nesting resources (i.e., wood cavities, coarse woody debris, and canopy cover) and floral resources (i.e., floral abundance and richness) per plot on nesting bee species richness, we used a generalized linear mixed‐effects model (GLMM) with a poisson distribution and included sampling block (*N* = 18) as a random effect to account for the nested study design. Furthermore, we tested for differences among nesting bee and floral community composition across burn severity (RdNBR) and time‐since‐burn (years postburn) with permutational multivariate analysis of variance (PERMANOVA) using the adonis function in the R package vegan (Oksanen et al., 2018). We then sought further detail into which floral species contributed most strongly to community composition differences across burn severities using a similarity percentage analysis (SIMPER, Oksanen et al., 2018).

To test for differences in nesting success with burn severity (RdNBR), time‐since‐burn (years postburn), the interaction between burn severity and time‐since‐burn, nesting resources (i.e., wood cavities, coarse woody debris, and canopy cover) and floral resources (i.e., floral abundance and richness), we used a Bayesian binomial GLMM approach with Markov chain Monte Carlo (MCMC) sampling at the nesting tube level. To account for the nested study design in which nesting tubes were nested within trap nests, within sampling blocks, and within fire perimeter we included sampling plot (i.e., bee nest box) within sampling block and fire perimeter as a nested random effect. We used this analytical approach to assess hypothesized drivers of nesting success because it computes group‐specific regression coefficients which have unknown covariance matrices, which was desirable as we had zero bee emergence at all unburned plots (see Section 3). Default, weakly informative priors from the rstanarm package version 2.18.2 were used (Goodrich, Gabry, Ali, & Brilleman, 2018), and we drew 2,000 samples each from four independent MCMC chains with the first 1,000 runs of each chain as model warm‐up and the latter half as effective sampling. This approach does not provide a significance test; instead, we assessed whether 95% posterior density intervals surrounding each mean parameter estimate overlapped zero. Posterior density intervals were calculated from posterior distribution draws with all four chains merged via the mcmc_intervals function in the bayesplot R package (Gabry & Mahr, 2018). These posterior density intervals identify where 95% of the marginal posterior parameter estimates lie (i.e., the likelihood that the estimated mean of the parameter of interest lies within the interval) and thus can be considered analogous to a 95% confidence interval in typical frequentist binomial GLMs.

In models of bee richness and nesting success, we accounted for multicollinearity among nesting and floral resource parameters by testing the variance inflation factor (VIF) for each parameter within those models. Among the explanatory variables (wood cavities, coarse woody debris, canopy cover, floral richness, and floral abundance), only canopy cover met the VIF threshold (James, Witten, Hastie, & Tibshirani, 2013) and was removed. No further model selection was performed as our aim was to test the effects of these parameters which were hypothesized a priori to influence bee richness and nesting success. In all models, we ln‐transformed time‐since‐burn to rescale this parameter. In the model for nesting bee richness, we ln‐transformed the number of wood cavities, volume coarse woody debris, and floral abundance to rescale variables.

All analyses were performed in R 3.5.1 using the base, car, lme4, lmerTest, bayesplot, rstanarm, and vegan packages (Bates, Maechler, Bolker, & Walker, 2015; Fox and Weisberg, 2019; Gabry & Mahr, 2018; Goodrich et al., 2018; Kuznetsova, Brockhoff, & Christensen, 2017; Oksanen et al., 2018; R Core Development Team, 2018).

## 3 RESULTS

We collected 645 total occupied nesting tubes of which 236 (36.6%) had successful bee emergence. A total of 676 adult bees emerged, representing 18 species (*Megachile lapponica*—402, *Hoplitis albifrons argentifrons*—129, *Hylaeus modestus*—32, *Hylaeus verticalis—*28, *Megachile relativa*—27, *Stelis montana*—9, *Hylaeus basalis*—9, *Osmia pusilla*—8, *Osmia juxta*—7, *Coelioxys moesta*—7, *Hoplitis fulgida fulgida*—6, *Ashmeadiella californica*—5, *Osmia lignaria propinqua*—2, *Megachile pugnata*—1, *Megachile centuncularis*—1, *Heriades carinatus*—1, *Hylaeus colorodensis*—1, and *Osmia* spp.—1; Appendix S1). Raw data of species and number of individuals in each nesting tube in mixed‐ and high‐severity plots in each wildfire are available in Simanonok (2018). No emergence was observed from any of the 49 occupied nesting tubes collected in our unburned sampling plots (Table 2).

**Table 2:** Number of occupied nesting tubes, number of nesting tubes with successful bee emergence, and percentage nesting success (successful bee emergence) for each wildfire as well as both severity treatments

Burn name (years postburn)	Severity	Occupied tubes	Successful tubes	Nesting success (%)
Emigrant (3)	High	35	15	42.9
	Mixed	63	49	77.8
Pine Creek (4)	High	76	30	39.5
	Mixed	56	18	32.1
Wicked Creek (9)	High	94	35	37.2
	Mixed	121	69	57.0
Thompson Creek (25)	High	68	17	25.0
	Mixed	83	3	3.6
Unburned (>75)	–	49	0	0
Total	–	645	236	36.6

### 3.1 Nesting and floral resources

**Figure 1:**
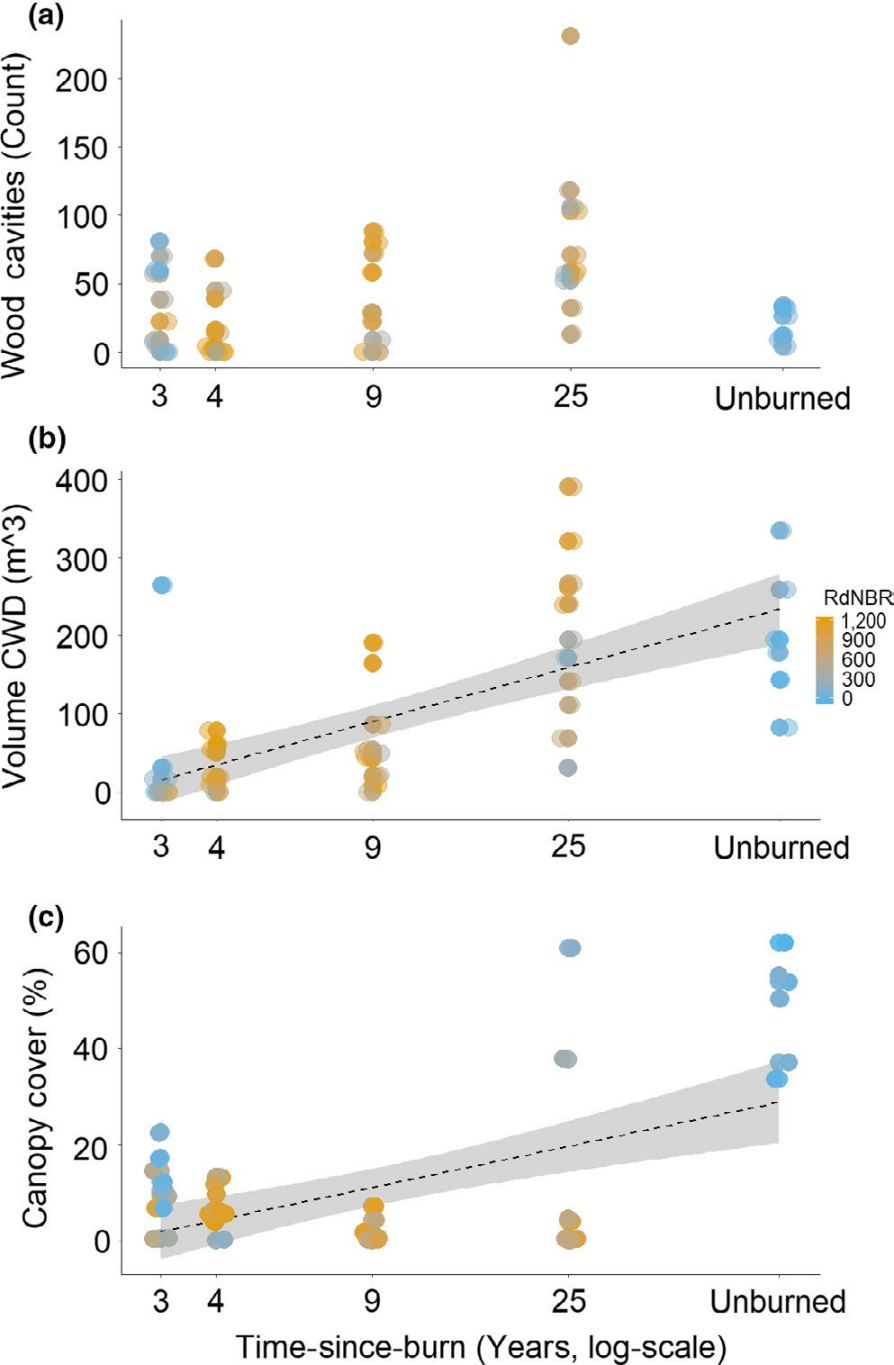
Number of wood cavities (a), volume coarse woody debris (CWD, b), and percent canopy cover (c) per sampling plot across years postburn and colored by burn severity (RdNBR). Trend lines indicate line of best fit with 95% confidence intervals for significant fits between parameter and time‐since‐burn. Points have been slightly faded and jittered to improve visibility. See Table 3 for model outputs

**Figure 2:**
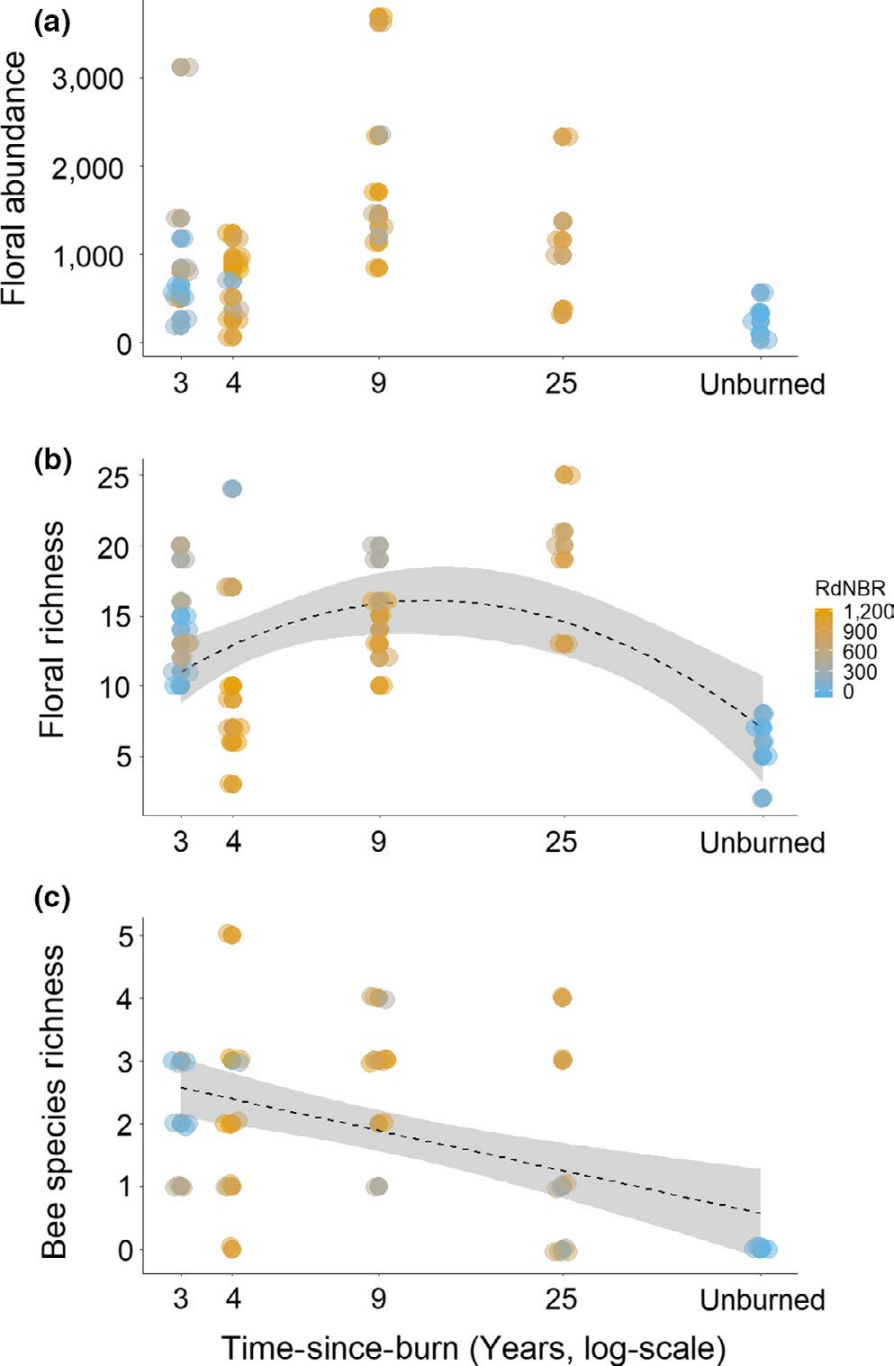
Floral abundance (a), floral richness (b), and bee species richness (c) per sampling plot plotted across years postburn and colored by burn severity (RdNBR). Trend lines indicate line of best fit with 95% confidence intervals for significant fits between parameter and time‐since‐burn. Points have been jittered to improve visibility. See Table 4 for model outputs

The number of wood cavities weakly declined with burn severity at greater years of time‐since‐burn, with the fewest wood cavities being observed in unburned sites (Table 3, Figure 1a). CWD was generally greater in older burns and unburned sites yet also in early, lower‐severity sites (Table 3, Figure 1b). Similarly, canopy cover was greatest in older burns, unburned sites, and early, low‐severity sites (Table 3, Figure 1c). There was no significant relationship between floral abundance and time‐since‐burn or burn severity (Table 4, Figure 2a). Floral richness varied nonlinearly with time‐since‐burn peaking at intermediately aged burn sites and being relatively lower at early, low‐severity as well as unburned sites (Table 4, Figure 2b). Floral community composition varied across time‐since‐burn, and this relationship depended upon burn severity (Table 5). *Chamerion angustifolium* and *Physocarpus malvaeceus* contributed most strongly to community dissimilarity by burn severity (Appendix S2).

**Table 3:** Multiple regression model results for number of wood cavities, volume coarse woody debris, and percent canopy cover

	Estimate	*SE*	*t*	*p*
Wood cavities				
Model intercept	3.64	0.53	6.81	**<.01**
Burn severity	−2.10e^−3^	8.61e^−4^	−2.44	**.02**
Time‐since‐burn	−0.09	0.18	−0.47	.64
Burn severity × Time‐since‐burn	1.04e^−3^	3.20e^−4^	3.23	**<.01**
Coarse Woody Debris				
Model intercept	−22.2	35.8	−0.62	.54
Burn severity	−0.13	0.06	−2.41	**.02**
Time‐since‐burn	45.1	12.2	3.70	**<.01**
Burn severity × Time‐since‐burn	0.08	0.02	3.28	**<.01**
Canopy cover				
Model intercept	−6.86	4.51	−1.52	.13
Burn severity	0.02	0.01	3.28	**<.01**
Time‐since‐burn	13.2	1.54	8.58	**<.01**
Burn severity × Time‐since‐burn	−0.02	2.90e^−3^	−6.83	**<.01**

Note

*p*‐Values are bolded at *α* < 0.05.

**Table 4:** Multiple regression model outputs for floral abundance and floral richness

	Estimate	*SE*	*t*	*p*
Floral abundance				
Model intercept	6.82	0.30	22.6	**<.01**
Burn severity	−1.35e^−4^	4.10e^−4^	−0.33	.74
Time‐since‐burn	−1.67	1.64	−1.02	.32
Time‐since‐burn^2^	−3.03	1.92	−1.58	.12
Burn severity × Time‐since‐burn	−3.72e^−4^	2.87e^−3^	−0.13	.90
Burn severity × Time‐since‐burn^2^	−2.23e^−3^	2.89e^−3^	−0.77	.44
Floral richness				
Model intercept	2.88	0.13	22.3	**<.01**
Burn severity	−2.86e^−4^	1.70e^−4^	−1.68	.10
Time‐since‐burn	−1.57	0.59	−2.67	**.01**
Time‐since‐burn^2^	−3.13	0.82	−3.83	**<.01**
Burn severity × Time‐since‐burn	4.12e^−3^	1.11e^−3^	3.72	**<.01**
Burn severity × Time‐since‐burn^2^	3.21e^−3^	1.36e^−3^	2.36	**.02**
Bee richness				
Model intercept	−0.07	1.08	−0.06	.95
Burn severity	−1.25e^−3^	8.91e^−4^	−1.40	.16
Time‐since‐burn	−0.83	0.35	−2.41	**.02**
Wood cavities	−0.14	0.06	−2.13	**.03**
CWD	4.80e^−3^	0.16	0.03	.98
Floral richness	0.01	0.03	0.46	.65
Floral abundance	0.31	0.16	1.94	**.05**
Burn severity × Time‐since‐burn	9.47e^−4^	4.87e^−4^	1.95	**.05**

Note

*p*‐Values are bolded at *α* < 0.05.

**Table 5:** Permutational multivariate analysis of variance results comparing the nesting bee community composition and floral community composition dissimilarities across burn severity, time‐since‐burn, and the interaction of burn severity and time‐since‐burn

	*df*	SS	Mean SS	*F*	*R* ^2^	*p*
Nesting bee community						
Burn severity	1	0.27	0.27	0.96	0.02	.48
Time‐since‐burn	1	0.39	0.39	1.39	0.03	.22
Burn severity × Time‐since‐burn	1	0.24	0.24	0.86	0.02	.54
Residuals	37	10.4	0.28		0.92	
Floral community						
Burn severity	1	0.35	0.35	0.98	0.02	.45
Time‐since‐burn	1	1.39	1.39	3.93	0.07	**<.01**
Burn severity × Time‐Since‐Burn	1	1.24	1.24	3.51	0.07	**<.01**
Residuals	44	15.5	0.35		0.84	

Note

*p*‐Values are bolded at *α* < 0.05.

### 3.2 Nesting bee species richness

Bee species richness declined ca. 0.8 species with each doubling of time‐since‐burn and declined with the number of wood cavities (Table 4, Figure 2c). However, bee richness increased with greater floral abundance (Table 4). The community composition of nesting bees did not change across burn severity or time‐since‐burn (Table 5).

### 3.3 Nesting bee emergence

**Figure 3:**
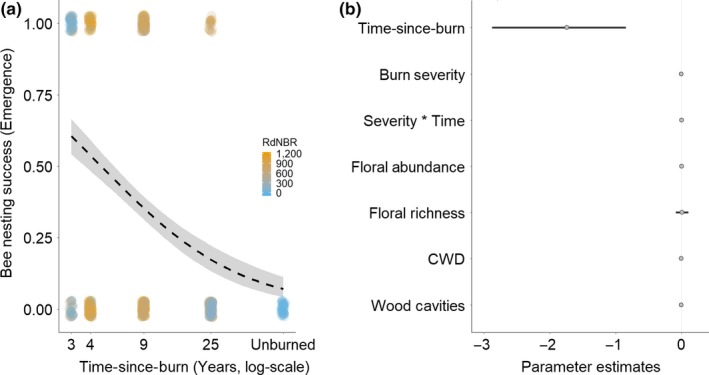
(a) Bee nesting success measured as emergence of individual nesting tubes over time‐since‐burn and colored by burn severity (RdNBR). Line represents significant line of best fit for binomial Bayesian generalized linear mixed‐effects model (GLMM). (b) Bayesian GLMM parameter estimates for the effects on bee nesting success measured as emergence. Points are parameter estimate means with 95% posterior density intervals as lines

Bee emergence declined by ca. 1.74% per year with increasing time‐since‐burn (Figure 3a,b). The mean parameter estimates for all other model parameters (burn severity, burn severity × time‐since‐burn, floral abundance, floral richness, coarse woody debris, and number of wood cavities) overlapped zero, providing insufficient evidence that bee emergence varies with those parameters (Figure 3b).

## 4 DISCUSSION

We investigated the effects of wildfire severity and time‐since‐burn on wood‐cavity‐bee nesting success and species richness postburn. Wood‐cavity‐nesting bee nesting success was greatest in the years immediately postburn and declined with increasing time‐since‐burn, highlighting the importance of early successional, postburn habitats for cavity‐nesting bees. Furthermore, we observed limited nesting success in older burns and no successful bee emergence from unburned plots, suggesting that bee reproduction would be minimal without burned areas; therefore, burned areas, regardless of burn severity, likely represent key nesting habitat for cavity‐nesting species in this system. We also observed declines in nesting bee species richness with increasing time postburn, suggesting that unburned areas may not be suitable nesting habitat for wood‐cavity‐nesting bee species we observed in burned areas. Reductions in bee nesting success and nesting bee species richness with greater time‐since‐burn are novel findings, are consistent with previously observed patterns of declining bee abundance with increasing time‐since‐burn in a different system (e.g., Potts et al., 2003), and may provide at least a partial explanation for why bee abundance declines with time‐since‐burn. Despite finding that several nesting and floral resources varied with burn severity and time‐since‐burn, we observed no relationships between measured nesting or floral resources with bee nesting success. However, we did observe bee richness to negatively correlate with number of wood cavities and positively correlate with floral abundance, suggesting that the richness of nesting bee communities can be limited by available nesting resources or enhanced by ample floral resources. Below we discuss the role of burn severity and early postburn habitats in supporting cavity‐nesting bee communities, why nesting success may decline through successional time, as well as the role of nesting and floral resources for wood‐cavity bee nesting in postburn ecosystems.

We observed nesting success to decline with increasing time‐since‐burn, with zero bee emergence at unburned sites, and this may provide some insight into the heterogeneity of resources across the postburn landscape. Most solitary bee taxa have relatively limited foraging ranges such that the probability of nesting drops sharply if adequate forage is greater than 250–600 m from the nest (Gathmann & Tscharntke, 2002), and bee diversity and abundance have been observed to respond to fire most strongly at those spatial scales (250–300 m, Lazarina et al., 2019). Thus, if the distance between foraging and nesting habitats is too great (e.g., Westrich, 1996), low nesting success is likely. Interestingly, this may be the case at our older burns and unburned sites, where there are ample nesting resources but relatively few floral resources. By contrast, at burned sites, there are abundant floral communities which are compositionally different from unburned sites, particularly at intermediately aged burns, and lower levels of nesting resources, yet nevertheless enough nesting resources to support some degree of bee nesting. However, within those burned areas we observed no effects of burn severity on nesting success, suggesting that successional postburn processes (i.e., time‐since‐burn) are more important for regulating wood‐cavity‐nesting bees than burn severity in this system. Taken together, these patterns could imply that floral resources are more limiting than nesting resources for the wood‐cavity‐nesting bees in this system and that maintaining landscape heterogeneity is key in the management and conservation of solitary cavity‐nesting bees after wildfire, to provide intermixed areas of nesting and floral resources.

We observed nesting resources to vary significantly across both burn severity and time‐since‐burn, and patterns in nesting resources for wood‐cavity‐nesting bees have been inconsistent across previous studies. One study found the number of natural wood cavities and wood cavity occupancy to increase with forest stand age postlogging, while trap nest use did not change with stand age for most taxa (however, cellophane‐like plugs, such as those made by *Hylaeus* spp. increased, Westerfelt et al., 2015), whereas others have found the number of wood cavities to increase with burn severity (Galbraith et al., 2019). We observed lower bee richness where there were fewer wood cavities, suggesting that the number of species nesting could be influenced by available nesting resources. Since wood‐cavity‐nesting bees are dependent upon cavities built by wood‐boring beetles, it is possible that wood‐cavity bee nesting habitat may be beetle‐limited at our unburned sites (sensu Sydenham et al., 2016), although we did not collect data to test this hypothesis. While CWD and canopy cover did increase with time‐since‐burn, neither correlated with bee species richness, and wood cavities are likely a more direct measure of available nesting habitat. Thus, while we did not observe nesting resources did to affect bee nesting success, nesting resources may limit the richness of bee species which are able to establish in an area.

This study is the first to directly record the species richness of the community of actively nesting bees in a postburn landscape, and we found a weak interactive effect of burn severity with greater time‐since‐burn, where richness was lowest in older, mixed‐severity burns, and unburned sites. Previous studies of foraging bee responses to burn severity have shown conflicting results. For example, bee diversity has been observed to increase with greater pyrodiversity in mixed‐conifer forest in the Sierra Nevadas (Ponisio et al., 2016) and bee richness has been observed to be greater in mixed‐ compared to high‐severity burns in this system (Simanonok, 2018); however, that finding was based on a larger range of bee species whereas this manuscript only focuses on a specific nesting guild. By contrast, other recent studies have found bee diversity to be greatest in moderate burn severities in Mediterranean pine forests (Lazarina et al., 2019) or in high‐severity burns for douglas fir‐dominated forests in Oregon (Galbraith et al., 2019), or to not differ between mixed‐ and high‐severity burns for several forest types across Montana (LaManna et al., in review). One potential explanation in this discrepancy across studies is the interactive effects of burn severity and time‐since‐burn; that is, the effects of burn severity can vary in both direction and magnitude across successional time. Furthermore, this high variability among studies in the direction of foraging bee richness and diversity responses to burn severity suggests that the effect of burn severity on bee communities is likely to be highly system‐ and potentially taxa‐specific as well.

Innovation in effectively sampling bee nesting habitat use in situ is sorely needed and could revolutionize our understanding of the relative importance of nesting and floral resources for bee species as well as the conditions under which either could be limiting. Currently, locating wild cavity‐nesting bees is exceptionally difficult (Westerfelt et al., 2015) and successful efforts have required extensive sampling effort, high levels of local entomological expertise, and even then yielded low detection rates for natural occupied cavities (ca. 1%, Westerfelt et al., 2015). It is important to note that bee nesting was only assessed for wood‐cavity‐nesting bees; we did not investigate other bee guilds, although experimental evidence suggests ground‐nesting bees are unlikely to be affected by wildfires, even those that burn with high‐severity (Cane & Neff, 2011). Future studies which sample nesting bees in situ will be necessary to understand the effects of nesting and floral resources on bee richness, abundance, and nesting success, particularly in disturbed landscapes.

In this system, we observed wood‐cavity bee nesting success and bee species richness to decline with increasing time‐since‐burn. For nesting and floral resources, as well as bee species richness, we also observed interactive effects of burn severity with time‐since‐burn, highlighting a need to consider burn severity in the context of succession in future studies. With the continued change of fire regimes coupled with other concurrent disturbances (Bowman et al., 2009; Potts et al., 2010) in systems globally, understanding the threats to wild bee species, particularly those with specific habitat requirements like wood‐cavity‐nesting bees, will be especially important for species conservation and management (Potts et al., 2010).

## CONFLICT OF INTEREST

The authors declare that they have no conflict of interests.

## AUTHOR CONTRIBUTIONS

MPS and LAB contributed to the concept and design of the study, MPS acquired the data and performed analyses, and MPS and LAB drafted the manuscript.

## Data Availability

Data used for analyses in this publication are stored in the Research Data Archive maintained by the US Forest Service and US Department of Agriculture. https://doi.org/10.2737/RDS-2019-0037
