# Supplementary material for: Nesting success of wood‐cavity‐nesting bees declines with increasing time since wildfire
Source: Ecol Evol. 2019 Oct 2;9(22):12436–45. doi: 10.1002/ece3.5657 (PMC6875575; doi:10.1002/ece3.5657)
Supplement: Supplementary file 1 [file ECE3-9-12436-s001.docx]

**Appendix 1.** Bee species emerged from nesting tubes including the number of individuals of each sex within each fire perimeter and each burn severity.

| **Species** | **Sex** | **Fire** | **Severity** | **# Individuals** |
| --- | --- | --- | --- | --- |
| *Ashmeadiella californica* | F | Wicked Creek | Mixed | 5 |
| *Coelioxys moesta* | F | Emigrant | Mixed | 1 |
| *Coelioxys moesta* | F | Pine Creek | High | 1 |
| *Coelioxys moesta* | F | Wicked Creek | High | 1 |
| *Coelioxys moesta* | M | Emigrant | Mixed | 3 |
| *Coelioxys moesta* | M | Thompson Creek | High | 1 |
| *Heriades carinatus* | F | Pine Creek | Mixed | 1 |
| *Hoplitis albifrons argentifrons* | F | Emigrant | High | 5 |
| *Hoplitis albifrons argentifrons* | F | Emigrant | Mixed | 1 |
| *Hoplitis albifrons argentifrons* | F | Pine Creek | High | 7 |
| *Hoplitis albifrons argentifrons* | F | Pine Creek | Mixed | 9 |
| *Hoplitis albifrons argentifrons* | F | Wicked Creek | High | 17 |
| *Hoplitis albifrons argentifrons* | F | Wicked Creek | Mixed | 22 |
| *Hoplitis albifrons argentifrons* | F | Thompson Creek | High | 7 |
| *Hoplitis albifrons argentifrons* | F | Thompson Creek | Mixed | 2 |
| *Hoplitis albifrons argentifrons* | M | Emigrant | High | 1 |
| *Hoplitis albifrons argentifrons* | M | Emigrant | Mixed | 3 |
| *Hoplitis albifrons argentifrons* | M | Pine Creek | High | 3 |
| *Hoplitis albifrons argentifrons* | M | Pine Creek | Mixed | 3 |
| *Hoplitis albifrons argentifrons* | M | Wicked Creek | High | 6 |
| *Hoplitis albifrons argentifrons* | M | Wicked Creek | Mixed | 41 |
| *Hoplitis albifrons argentifrons* | M | Thompson Creek | High | 2 |
| *Hoplitis fulgida fulgida* | F | Thompson Creek | High | 4 |
| *Hoplitis fulgida fulgida* | M | Thompson Creek | High | 2 |
| *Hylaeus basalis* | F | Emigrant | High | 3 |
| *Hylaeus basalis* | M | Emigrant | High | 6 |
| *Hylaeus colorodensis* | F | Emigrant | High | 1 |
| *Hylaeus modestus* | F | Emigrant | Mixed | 3 |
| *Hylaeus modestus* | F | Pine Creek | High | 4 |
| *Hylaeus modestus* | F | Wicked Creek | Mixed | 2 |
| *Hylaeus modestus* | F | Thompson Creek | High | 2 |
| *Hylaeus modestus* | M | Emigrant | High | 2 |
| *Hylaeus modestus* | M | Emigrant | Mixed | 8 |
| *Hylaeus modestus* | M | Pine Creek | High | 1 |
| *Hylaeus modestus* | M | Wicked Creek | Mixed | 3 |
| *Hylaeus modestus* | M | Thompson Creek | High | 7 |
| *Hylaeus verticalis* | F | Emigrant | High | 9 |
| *Hylaeus verticalis* | F | Emigrant | Mixed | 1 |
| *Hylaeus verticalis* | F | Wicked Creek | Mixed | 1 |
| *Hylaeus verticalis* | F | Thompson Creek | Mixed | 2 |
| *Hylaeus verticalis* | M | Emigrant | High | 9 |
| *Hylaeus verticalis* | M | Wicked Creek | Mixed | 1 |
| *Hylaeus verticalis* | M | Thompson Creek | Mixed | 5 |
| *Megachile centuncularis* | M | Wicked Creek | High | 1 |
| *Megachile lapponica* | F | Emigrant | Mixed | 31 |
| *Megachile lapponica* | F | Pine Creek | High | 16 |
| *Megachile lapponica* | F | Pine Creek | Mixed | 4 |
| *Megachile lapponica* | F | Wicked Creek | High | 6 |
| *Megachile lapponica* | F | Wicked Creek | Mixed | 14 |
| *Megachile lapponica* | F | Thompson Creek | High | 12 |
| *Megachile lapponica* | M | Emigrant | High | 5 |
| *Megachile lapponica* | M | Emigrant | Mixed | 143 |
| *Megachile lapponica* | M | Pine Creek | High | 36 |
| *Megachile lapponica* | M | Pine Creek | Mixed | 32 |
| *Megachile lapponica* | M | Wicked Creek | High | 22 |
| *Megachile lapponica* | M | Wicked Creek | Mixed | 65 |
| *Megachile lapponica* | M | Thompson Creek | High | 16 |
| *Megachile pugnata* | F | Pine Creek | High | 1 |
| *Megachile relativa* | F | Pine Creek | High | 5 |
| *Megachile relativa* | F | Pine Creek | Mixed | 1 |
| *Megachile relativa* | F | Wicked Creek | High | 5 |
| *Megachile relativa* | F | Wicked Creek | Mixed | 1 |
| *Megachile relativa* | M | Pine Creek | High | 7 |
| *Megachile relativa* | M | Pine Creek | Mixed | 2 |
| *Megachile relativa* | M | Wicked Creek | High | 3 |
| *Megachile relativa* | M | Wicked Creek | Mixed | 3 |
| *Osmia juxta* | F | Wicked Creek | High | 7 |
| *Osmia lignaria propinqua* | F | Emigrant | High | 1 |
| *Osmia lignaria propinqua* | M | Emigrant | High | 1 |
| *Osmia pusilla* | F | Emigrant | Mixed | 2 |
| *Osmia pusilla* | M | Emigrant | Mixed | 6 |
| *Osmia sp1* | M | Pine Creek | High | 1 |
| *Stelis montana* | F | Emigrant | High | 1 |
| *Stelis montana* | F | Pine Creek | High | 3 |
| *Stelis montana* | F | Wicked Creek | High | 1 |
| *Stelis montana* | F | Wicked Creek | Mixed | 3 |
| *Stelis montana* | F | Thompson Creek | Mixed | 1 |

| **High vs. Mixed** | |
| --- | --- |
| **Species** | **Contribution** |
| *Chamerion angustifolium* | 0.18 |
| *Physocarpus malvaceus* | 0.27 |
| *Symphoricarpos albus* | 0.34 |
| *Antennaria racemosa* | 0.4 |
| *Spiraea betulifolia* | 0.44 |
| *Arnica cordifolia* | 0.48 |
| *Achillea millefolium* | 0.51 |
| *Lupinus sericeus* | 0.55 |
| *Galium boreale* | 0.58 |
| *Anaphalis margaritacea* | 0.61 |
| *Linnaea borealis* | 0.63 |
| *Campanula rotundifolia* | 0.65 |
| *Ceanothus velutinus* | 0.68 |
| *Thalictrum occidentale* | 0.7 |
| *Mahonia repens* | 0.72 |
| **High vs. Unburned** | |
| **Species** | **Contribution** |
| *Chamerion angustifolium* | 0.2 |
| *Physocarpus malvaceus* | 0.36 |
| *Lupinus sericeus* | 0.44 |
| *Spiraea betulifolia* | 0.51 |
| *Antennaria racemosa* | 0.57 |
| *Hedysarum sulphurescens* | 0.62 |
| *Achillea millefolium* | 0.68 |
| *Symphoricarpos albus* | 0.72 |
| **Mixed vs. Unburned** | |
| **Species** | **Contribution** |
| *Chamerion angustifolium* | 0.28 |
| *Physocarpus malvaceus* | 0.4 |
| *Spiraea betulifolia* | 0.49 |
| *Lupinus sericeus* | 0.56 |
| *Hedysarum sulphurescens* | 0.64 |
| *Achillea millefolium* | 0.69 |
| *Astragalus agrestis* | 0.72 |

**Appendix 2:** Similarity percentages results from the simper function in the R package vegan. Cumulative contributions of the most influential species affecting species composition groupings between burn severities (High, Mixed, and Unburned).
